# A Subset of Microsatellite Unstable Cancer Genomes Prone to Short Insertions over Deletions Is Associated with Elevated Anticancer Immunity

**DOI:** 10.3390/genes15060770

**Published:** 2024-06-12

**Authors:** Sunmin Kim, Dong-Jin Han, Seo-Young Lee, Youngbeen Moon, Su Jung Kang, Tae-Min Kim

**Affiliations:** 1 Department of Medical Informatics, College of Medicine, The Catholic University of Korea, Seoul 06591, Republic of Korea; smkim46@catholic.ac.kr (S.K.);; 2 Cancer Research Institute, College of Medicine, The Catholic University of Korea, Seoul 06591, Republic of Korea; 3 Department of Biomedicine & Health Sciences, Graduate School, The Catholic University of Korea, Seoul 06591, Republic of Korea; 4 CMC Institute for Basic Medical Science, The Catholic Medical Center, The Catholic University of Korea, Seoul 06591, Republic of Korea

**Keywords:** microsatellite instability, DNA repair, indel, mutational signature, anticancer immunity

## Abstract

Deficiencies in DNA mismatch repair (MMRd) leave characteristic footprints of microsatellite instability (MSI) in cancer genomes. We used data from the Cancer Genome Atlas and International Cancer Genome Consortium to conduct a comprehensive analysis of MSI-associated cancers, focusing on indel mutational signatures. We classified MSI-high genomes into two subtypes based on their indel profiles: deletion-dominant (MMRd-del) and insertion-dominant (MMRd-ins). Compared with MMRd-del genomes, MMRd-ins genomes exhibit distinct mutational and transcriptomic features, including a higher prevalence of T>C substitutions and related mutation signatures. Short insertions and deletions in MMRd-ins and MMRd-del genomes target different sets of genes, resulting in distinct indel profiles between the two subtypes. In addition, indels in the MMRd-ins genomes are enriched with subclonal alterations that provide clues about a distinct evolutionary relationship between the MMRd-ins and MMRd-del genomes. Notably, the transcriptome analysis indicated that MMRd-ins cancers upregulate immune-related genes, show a high level of immune cell infiltration, and display an elevated neoantigen burden. The genomic and transcriptomic distinctions between the two types of MMRd genomes highlight the heterogeneity of genetic mechanisms and resulting genomic footprints and transcriptomic changes in cancers, which has potential clinical implications.

## 1. Introduction

Impairment of the genome maintenance system results in genomic instability, which is characterized by an accumulation of various types of genomic abnormalities. This phenomenon is particularly evident in certain cancer genomes that exhibit frequent insertions and deletions (indels) at microsatellites due to a deficiency in DNA mismatch repair (MMRd), which is also associated with high mutation rates [1]. This hypermutability, known as microsatellite instability (MSI), occurs due to DNA slippage or mismatches that occur during the replication of short repeated DNA sequences known as microsatellites [2]. Although such errors are typically corrected by DNA mismatch repair (MMR) in normal cells, genomes with MMRd show MSI, which occurs as either a decrease or increase in the repeated units within microsatellite sequences. The first observation of MSI, characterized as ‘ubiquitous somatic mutations at simple repeated sequences’, in this case deletions involving adenine residues, was reported by Ionov et al. [3]. Their finding was confirmed by Thibodeau et al. [4], who noted frequent deletions in microsatellite sequences containing [CA]n repeats [4,5]. Although microsatellite instability-high (MSI-H) status is often associated with certain hereditary cancer syndromes such as Lynch syndrome (hereditary non-polyposis colorectal cancer) [6], most MSI-H cases arise sporadically. Germline and somatic alterations in MMR pathway genes have been reported, including hypermethylation of the *MLH1* gene promoter, and inherited germline mutations in MMR genes are frequent, including in *MLH1*, *MSH2*, *MSH6*, and *PMS2* [7,8]. MSI is prevalent in colorectal carcinoma (CRC) and uterine corpus endometrial carcinoma (UCEC) and has also been observed in stomach, ovarian, cervical, esophageal, skin, and breast cancers [9,10]. MSI has been linked to the tumor mutation burden (TMB) in cancer genomes and recognized as a tumor type–agnostic biomarker for immune checkpoint blockade, indicating that it influences cancer prognosis and treatment [11,12,13,14].

Mutational signatures, as genomic footprints that correspond to various mutagenic processes in the DNA replication and repair pathways, can be identified from the mutation profiles of cancer genomes [15,16]. Mutational signatures, which cover genetic alterations from single base substitution (SBSs) and indels (IDs) to chromosomal-level copy number alterations and structural variations, delineate the types and magnitudes of individual mutagenic processes active in cancer genomes. In addition to well-recognized smoking- and ultraviolet-related signatures (SBS4 and SBS7, respectively, in the annotation of the COSMIC v3 database) [17], DNA repair or proofreading deficiencies generate their own cognate footprints, such as SBS3 (homologous recombination deficiency) and SBS10 (polymerase proofreading deficiency) [17]. Several signatures, such as SBS6, SBS14, SBS15, SBS20, SBS21, SBS26, and SBS44, have been reported to be associated with MMRd [17]. Although they are less understood than SBS mutation signatures, the ID1 and ID2 indel signatures are assumed to be associated with MMRd [17].

Mutation signature-based analyses have demonstrated that DNA mutagenic and repair processes have a peculiar relationship. For example, it is unclear whether MMRd genomes are largely exclusive of other types of genomic instability, e.g., deficiencies in polymerase proofreading functions (PPd) with loss-of-function mutations in *POLD1* and *POLE*. However, PPd genomes are often accompanied by MMRd [18,19]. With intact MMR functions, *POLD1* and *POLE* mutations contribute to elevated mutation rates, but cancer genomes with combined MMRd and PPd tend to show a higher ID burden than those with only MMRd [1].

Despite the clinical implications of MSI genomes, previous research has focused on SBS mutation signatures. Few efforts have been made to examine MSI cancer genomes in terms of the ID mutation signatures that are the major genomic features of MMRd genomes. For this study, we conducted research using indel signatures as the primary measure for classifying cancer genomes. Our results showed that traditional MSI-H genomes can be classified into two types based on a preference for insertions or deletions. Although deletion-prone MMRd genomes account for most MSI-H genomes (~80%), insertion-prone MMRd genomes have unique mutational and transcriptomic properties.

## 2. Materials and Methods

### 2.1. Data Collection

We obtained somatic single-nucleotide variant (SNV) and indel profiles from whole-exome sequencing of colorectal cancer and endometrial cancer from the Cancer Genome Atlas (TCGA) consortium [20]. MSI status, RNA-seq profiles, copy number profiles, and methylation profiles (consensus of 27K and 450K profiles) were obtained from cBioPortal [21] (https://www.cbioportal.org/, accessed on 4 May 2023). TCGA pathogenic germline variant profile (https://gdc.cancer.gov/about-data/publications/PanCanAtlas-Germline-AWG, accessed on 31 July 2023) and neoantigen predictions (https://gdc.cancer.gov/about-data/publications/panimmune, accessed on 31 October 2023) were obtained from the Genome Data Commons portal [22]. Somatic mutation, MSI, and structural variant profiles from whole-genome sequencing were obtained from the International Cancer Genome Consortium (ICGC) Pan-Cancer Analysis of Whole Genomes (PCAWG) [20,23,24] (https://dcc.icgc.org/releases/PCAWG, accessed on 29 June 2023). We calculated the TMB using the maftools R package (version 2.2.10) [25].

### 2.2. Mutational Signature Analysis

From an initial dataset of 528 samples of CRC and 525 samples of UCEC, we applied a filtering criterion to exclude samples with fewer than the 10 indels that MSI-H status requires [26], resulting in 119 CRC and 221 UCEC samples. Subsequently, we selected 72 CRC and 189 UCEC samples that were either annotated as MSI-H or harboring *POLE/POLD1* exonuclease mutations. In the PCAWG data, only samples harboring more than 1000 indels were used, applying the same strategy used for the TCGA data. For the ID mutational signature analysis, we generated a mutational catalog comprising 83 indel features for each sample. This was accomplished using the YAPSA R package (version 1.24.0) [27] with reference to the 18 GRCh37 COSMIC ID signatures (ver. 3, 2019) [15,17].To identify the SBS signature contribution in each group, SNVs were assigned using 96 nucleotide triplets in the deconstructSigs (version 1.8) [28] and BSgenome.Hsapiens.UCSC.hg19 (version 1.4.0) [29] R packages. The reference signature was the GRCh37 COSMIC SBS signature (ver. 3, 2019) [15,17]. Cosine similarities were calculated using the maftools R package (version 2.2.10) on the median value of the SBS signature spectra for each group [25].

### 2.3. Genes Differentially Targeted and Expressed between MMRd-ins and MMRd-del Genomes

To observe the gene-level indel landscape, somatically truncated insertions and deletions present in the coding region were used. The levels of differential enrichment between the MMRd-ins and MMRd-del genomes were estimated at a significance level of *p*-value < 0.05 using Fisher’s exact test. To observe the SNV landscape, coding variants were similarly analyzed using Fisher’s exact test to determine their differential enrichment between the MMRd-ins and MMRd-del genomes.

To detect differentially expressed genes (DEGs) between MMRd and MMRd-ins, the t-test was used to determine p-values and fold-changes. Genes with a *p*-value < 0.05 and fold-change > 1 were selected as DEGs. A gene set enrichment analysis (GSEA) was performed with 1000 permutations using the fgsea R package (version 2.12) [30]. The Hallmark gene set from the human MSigDB gene set [31,32,33] was used as the signature database, and pathways with an adjusted p-value (False discovery rate; FDR) < 0.05 were deemed to be significantly enriched. The immune scores for all samples were calculated using ESTIMATE (version 1.10.13) [34], and tumor immune dysfunction and exclusion (TIDE) scores [35] were calculated using TIDEpy (https://github.com/jingxinfu/TIDEpy, accessed on 23 February 2024). For the immune cell deconvolution analysis, we used CIBERSORTx [36] with 22 immune cell types (LM22) as the signature matrix. To ensure the reliability of the *p*-value estimates, we conducted 100 permutations.

### 2.4. Identification of Neoantigens

We computed the number of samples expressing genes with strong (below 50 nM) Human Leukocyte Antigen (HLA) binding affinity for peptides in all mutations and calculated the sample proportions for each subtype. The structural variant (SV)-derived neoantigens in the PCAWG data were obtained from [37].

### 2.5. Structural Variations

To identify SVs, we initially grouped them into clusters and footprints using ClusterSV [38] (https://github.com/cancerit/ClusterSV, accessed on 17 April 2024) and then separated out the complex SVs. The remaining simple SVs (i.e., deletion, tandem duplication, inversion, and translocation) were subclassified into SV signatures based on previous descriptions [38]. The classification of deletions and tandem duplications was based on variant size using the following categories: 0 to 1 Mb as small, 1 Mb to 10 Mb as mid-sized, and >10 Mb as large. Inversions and translocations were classified as reciprocal inversions, fold-back inversions, reciprocal translocations, or unbalanced translocations. Clustered complex SVs were classified into six complex signatures using Starfish [39] (https://github.com/yanglab-computationalgenomics/Starfish, accessed on 23 April 2024).

## 3. Results

### 3.1. Indel-Based Classification of MMRd Genomes

**Figure 1:**
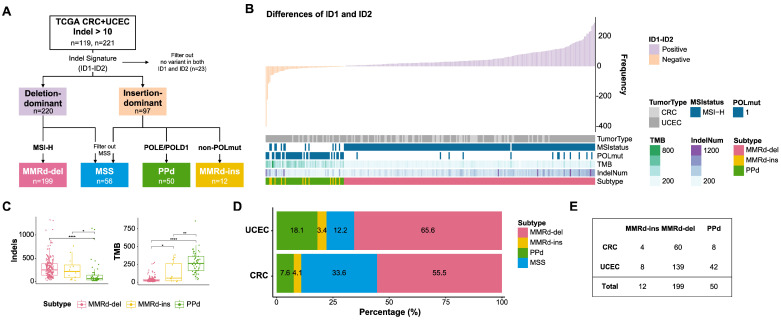
Distinct indel (ID) signatures and classification of subtypes. (**A**) Analysis workflow of subtype classification: A total of 119 and 221 individuals with an indel abundance greater than 10 were selected from the TCGA CRC and UCEC datasets, respectively. They were classified as deletion- or insertion-dominant indel signatures according to the frequency of ID1 and ID2, and 23 individuals with no frequency were filtered out. In that way, three subtypes were classified based on MSI-H status and the presence of *POLE*/*POLD1* mutations, excluding microsatellite stable genomes. (**B**) The top bar plot represents the indel frequency sorted by the difference in abundance between ID1 and ID2. The bottom bar represents the clinical information. (**C**) The tumor mutation and indel burden in each subtype: In MMRd-ins, both the tumor mutation and indel burdens were observed to be at intermediate levels in all three subtypes. * *p* < 0.05; ** *p* < 0.01; **** *p* < 0.0001. (**D**,**E**) Distribution of subtypes in both CRC and UCEC.

We obtained CRC and UCEC cancer genomes enriched in indel mutations (>10 indels per exome, >0.2 indels per Mb) from TCGA, representing 22.5% and 42.9% of these tumor types, respectively. These two tumor types were selected due to their known association with MSI, as characterized by MMRd and hypermutations of *POLD1* and *POLE* that lead to deficiencies in PPd. The categorization of these genomes based on their indels is outlined in Figure 1A. To classify the genomes, we used genomic features to annotate the ID (indel) mutational signatures, including the length of the associated repeat units (C/T for homopolymers and repeat length otherwise), the length of the indels, and the presence of microhomology. Figure S1A provides an overview of these indel features, highlighting 1 bp insertions and 1 bp deletions within homopolymers longer than 6 bp as the most prominent features, which are also known as the hallmarks of the ID1 and ID2 mutational signatures, respectively. Thus, MSI describes most of the indels in the genomes of these two types.

We next performed an ID mutational signature analysis by estimating the abundance of known ID mutational signatures by linear regression [28]. Hereafter, we use the SBS1–SBS85 (single base substitution) and ID1–ID18 signatures in accordance with the annotations in COSMIC mutation signature ver. 3. Two major ID mutation signatures (ID1 for deletions and ID2 for insertions) distinguished the genomes into deletion-dominant (*n* = 220) and insertion-dominant (*n* = 97), respectively. To indicate the causality of the indels, we also classified them as either MMRd (MSI-H) or PPd (exonuclease hotspot mutations in *POLD1* and *POLE*) genomes, excluding microsatellite stable genomes. Our findings indicate that the deletion-dominant genomes predominantly belonged to the MMRd category (MMRd-del, *n* = 199). In contrast, the insertion-dominant genomes included both PPd genomes (*n* = 50) and MMRd genomes (MMRd-ins, *n* = 12). The MMRd-ins genomes thus underwent an additional examination to confirm the absence of any functional *POLD1* and *POLE* mutations.

Figure 1B illustrates the examined genomes sorted in order of differences in ID1 and ID2 abundance and shown with MMRd and PPd annotations and other clinicopathological features (Figure 1B). We observed that the ID2 mutation signature corresponding to 1 bp insertions occurred predominantly in cases with *POLD1* and *POLE* mutations, whereas deletions were mainly associated with MMRd-del genomes. The number of indels and the TMB (the number of all of the non-silent mutations) were significantly elevated in tumors abundant in the ID2 and ID1 mutation signatures, respectively (*p* = 7.6 × 10^10^ and *p* = 2.3 × 10^23^, Wilcoxon test; Figure S1B). Consistently, the number of indels was notably higher in the MMRd-del and MMRd-ins genomes, whereas the TMB was the highest in the PPd genomes (Figure 1C). We also noted that the MMRd-ins genomes represent a minor fraction of MSI-H cases, but they are consistently present across tumor types, accounting for 3.4% (4 out of 119 cases) of CRC and 4.1% (8 out of 221 cases) of UCEC genomes (Figure 1D,E).

### 3.2. Mutational Signatures and Concordance between Subtypes

**Figure 2:**
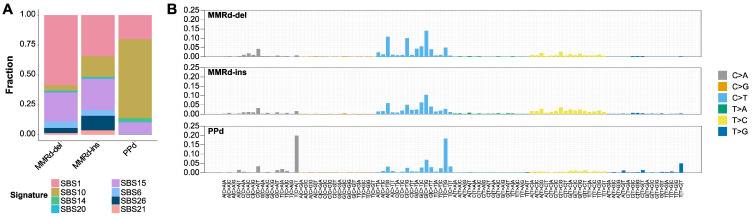
Distinct single base substitution (SBS) signatures across three subtypes. (**A**) Proportion of SBS signatures associated with MMRd and PPd. PPd has the highest proportion of SBS10 (brown), whereas MMRd-ins and MMRd-del are generally similar, with differences in the proportions of SBS1 (pink) and SBS26 (navy). (**B**) Mutational signature of each subtype. The *x*-axis represents the features of each nucleotide substitution, and the *y*-axis represents the proportions of the features.

We next analyzed the SBS mutation signatures, focusing on SBS6, SBS15, SBS21, and SBS26, which are linked to MMRd (SBS44 was excluded due to its low prevalence; see Table S1 for details about abundance across all of the SBS and ID mutation signatures examined). We also examined SBS10, which is linked to PPd, along with SBS14 and SBS20, which are recognized for their association with genomes exhibiting dual deficiencies in MMRd and PPd [17,26]. We observed a higher proportion of SBS10 in the PPd genomes, whereas the MMRd-related SBS mutation signatures were prevalent in the MMRd-del and MMRd-ins genomes. Although the SBS mutation signature profiles of the MMRd-del and MMRd-ins genomes were largely similar, they exhibited differences in the abundance of the SBS1 and SBS26 mutational signatures (Figure 2A). A statistical analysis further revealed significant differences in seven of the SBS mutation signature abundances across the MMRd-ins, MMRd-del, and PPd genome subtypes (Figure S2A).

We also analyzed the subtype-wise average abundance of 96 trinucleotide features corresponding to the SBS mutation signatures (Figure 2B). We observed that PPd genomes displayed mutational features related to SBS10 (e.g., T[C>A]T and T[C>T]C substitutions). Compared with the MMRd-del genomes, the MMRd-ins genomes exhibited more frequent T>C substitutions (Figure 2B and Figure S2B). Furthermore, subtype-specific mutational features were examined using cosine similarity to determine their correlation levels with known SBS mutation signatures. Among the mutational features of the PPd genomes, only the SBS10 mutation signature showed a high cosine similarity score of 0.77. Both the MMRd-del and MMRd-ins genomes showed elevated cosine similarity scores with MMRd-related signatures, particularly for SBS6 (0.94 and 0.88, respectively) and SBS15 (0.83 and 0.91, respectively). In comparing the MMRd-del and MMRd-ins genomes, the SBS1 mutational signature displayed lower cosine similarity scores with the MMRd-ins mutation features (0.83 vs. 0.71 with MMRd-del and MMRd-ins genomes, respectively). Notably, the MMRd-ins genomes also showed diminished cosine similarity with the SBS26 mutation signatures compared with the MMRd-del genomes (0.32 vs. 0.25 with MMRd-ins and MMR-del genomes, respectively) (Table S2). Thus, in spite of the overall similarity of the MMRd-related mutation signatures in the MMRd-del and MMRd-ins genomes, some mutation signatures (such as SBS26) can be used to distinguish between them.

### 3.3. Mutational Landscape of MMRd-ins, MMRd-del, and PPd Genomes

**Figure 3:**
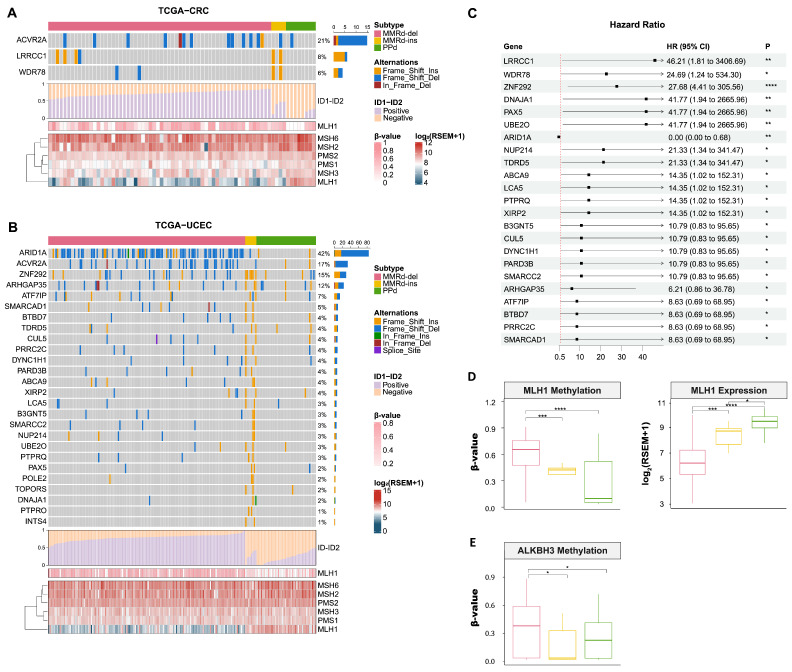
Landscape of target indels and MMR genes. Difference in somatic truncated indel profiles between the MMRd-ins and MMRd-del subtypes in (**A**) CRC and (**B**) UCEC: Target genes were selected based on a *p*-value < 0.05 in Fisher’s exact test. The bar plot in the middle represents the ratio of ID1 and ID2 in each sample. A heatmap of *MLH1* gene methylation and the expression of six MMR genes are displayed at the bottom. (**C**) Hazard ratios (HRs) of MMRd-ins target genes in both CRC and UCEC: The horizontal lines represent the 95% confidence intervals, and the square dots represent HR estimates. The red dashed line represents the reference value. *ARID1A* is identified as a significant gene in MMRd-del, with an HR value indicating 0. (**D**) *MLH1* gene methylation and expression in the three subtypes (**E**) Hypomethylation of the DDR-related *ALKBH3* gene in the MMRd-ins subtype; * *p* < 0.05; ** *p* < 0.01; *** *p* < 0.001; **** *p* < 0.0001.

We examined the gene-level consequences of the subtype-specific genomic variants (Figure 3A,B). Although no substantial differences in SBS frequencies were observed between the MMRd-ins and MMRd-del genomes (Figure S3A,B), the indels exhibited subtype-specific patterns (insertions and deletions in orange and blue, respectively; Figure 3A). For example, deletions in the *ACVR2A* gene, a well-recognized recurrent frameshift in MMRd genomes [40], were exclusively observed in the MMRd-del subtype (Figure 3A,B). *ARID1A* indels are common in tumor types with MSI-H and are known to suppress the function of tumor suppressor genes [41]. The observation that *ACVR2A* is subject to deletions in MMRd-del genomes but not to insertions in MMRd-ins genomes implies a unique vulnerability of this gene to specific types of indels.

Among MMRd-ins genomes, 2 and 25 genes in CRC and UCEC, respectively, were found to have significant insertions. Among them, *CUL5* and *SMARCAD1* belong to DNA damage repair (DDR) pathways, participating in nucleotide excision repair and homology-dependent recombination, respectively [42,43]. Frameshifting deletions of *ZNF292* and *SMARCC2* have been reported in gastric cancer and CRC with MSI, and they could play a role in the inactivation of tumor suppressor genes and tumorigenesis, respectively [44,45]. Figure 3C shows the genes differentially mutated between the MMRd-del and MMRd-ins subtypes in terms of hazard ratios, highlighting a significant difference for *ARID1A* (*p* = 8.5 × 10^3^, Fisher’s exact test). Although the analysis is limited by the small number of MMRd-ins genomes, these findings suggest that MMRd-del and MMRd-ins genomes exhibit distinct indel profiles, with their targeted genes being preferentially affected by deletions and insertions, respectively.

We next examined potential MSI-causal variants across the subtypes, focusing on genes in the MMR pathway. The level of promoter methylation of the *MLH1* gene was highest in the MMRd-del subtype, whereas that in the MMRd-ins genomes was intermediate between those of the MMRd-del and PPd genomes. DNA methylation is known to correlate with gene expression [46], and concordant with this, the expression levels of the *MLH1* gene were found to vary across the three subtypes (Wilcox rank-sum test; Figure 3D). As additional candidate DDR-related genes, the *ALKBH3* gene, which is associated with direct repair, exhibited higher promoter methylation in MMRd-del than MMRd-ins genomes (Figure 3E). Hypermethylation of the *ALKBH3* gene, compared with unmethylated status, has been reported to correlate with a worse prognosis in Hodgkin lymphoma [47]. Although the tumor type specificity of MMRd genomes that lead to hypermutable phenotypes has been partly explained by haploinsufficiency [48], our findings suggest that the differential dosage of *MLH1* and other DDR genes might also determine which types of MMRd genomes are particularly prone to specific types of indels. We were not able to identify significant differences across the three subtypes in the frequency of pathogenic germline variants in MMR genes (Figure S4A).

We next examined the copy number profiles of each subtype. In CRC, the MMRd-ins subtype had the lowest proportion of copy number variations among the subtypes (Figure S4B). Likewise, in UCEC, the MMRd-ins genomes showed relatively few copy number changes (Figure S4C). Although the actual number of somatic copy number alteration fragments remained constant (Figure S4D), genomes exhibiting a log2 difference of 0.1 or greater were observed in 22.6% and 31.8% of CRC and UCEC cases, respectively (Figure S4C). MMRd genomes are known to have relatively few copy number changes [49], and our findings indicate that this association is significant for the MMRd-ins subtype.

In addition, the MMRd-ins genomes exhibited a significantly lower cancer cell fraction (CCF) than the other subtypes for both SBSs and indels (Figure S4E). The CCF is used as a clonality measure, with low CCF values indicating subclonal mutations [50]. Thus, our results suggest that the mutations in the MMRd-ins genomes, including indels, are subclonal and fixed.

### 3.4. Comparing Gene Expression between MMRd-del and MMRd-ins Genomes

**Figure 4:**
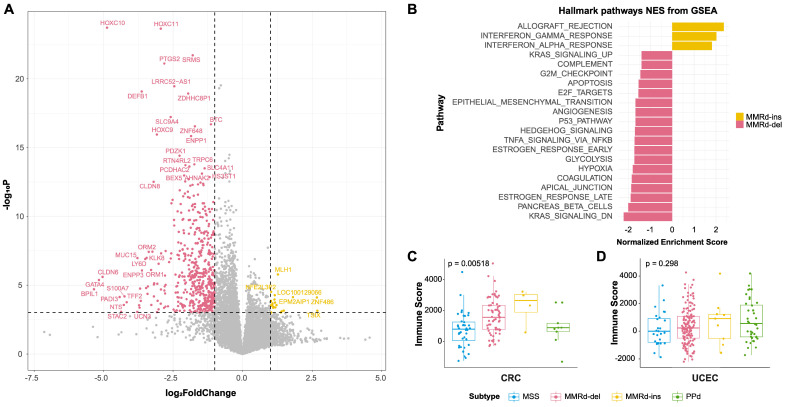
Differentially expressed genes (DEGs) and pathways between MMRd-del and MMRd-ins. (**A**) Volcano plot of DEGs between MMRd-ins and MMRd-del: The dashed lines represent the thresholds for the fold change (log2 fold change > 1) and *p*-value (<0.05). (**B**) Gene set enrichment analysis (GSEA) showing the top-ranked Hallmark pathways significantly altered (FDR < 0.05) in MMRd-ins versus MMRd-del. A positive normalized enrichment score (NES, orange) indicates enrichment in MMRd-ins, and a negative NES (pink) indicates enrichment in MMRd-del. (**C**) Immune scores from ESTIMATE in CRC and (**D**) UCEC: When compared with ANOVA, the *p*-values were 0.00518 and 0.298 for CRC and UCEC, respectively.

A DEG analysis was performed to compare the expression differences between the MMRd-ins and MMRd-del transcriptomes (see Methods). In the MMRd-del subtype, 521 genes exhibited significant differential expression, whereas in the MMRd-ins subtype, 21 genes showed significant differential expression. Within the MMRd-ins subtype, we observed significant differential expression of the *MLH1* gene, which is of particular importance due to its association with MSI and DNA mismatch repair processes (Figure 4A).

We further performed a GSEA to investigate the molecular functions transcriptionally enriched in each subtype. Immune-related functional pathways, such as genes belonging to the molecular terms of allograft rejection and interferon α/γ responses, were upregulated in MMRd-ins compared with MMRd-del (Figure 4B and Table S3). MMRd-del transcriptomes are relatively enriched in cancer hallmark functions such as G2M checkpoints and the epithelial-to-mesenchymal transition, indicative of their higher proliferative and invasive potential.

In addition, the immune ESTIMATE score [34] exhibited the highest tendency in MMRd-ins (ANOVA; Figure 4C,D). The TIDE score [35], which is a measurement of tumor immune escape, was also higher in MMRd-ins than MMRd-del (Figure S5A,B). The differential level of immunity-related scores among the groups were more pronounced in CRC than UCEC, indicative of tumor type specificity.

To further explore the immune cell composition in the tumor microenvironments (TMEs), we used immune cell deconvolution algorithms with 22 immune cell signatures, based on a support vector regression via CIBERSORTx [51]. The results revealed that the TME in the MMRd-ins subtype showed elevated infiltration of immune cells such as resting mast cells and memory B cells (Figure S5C). Thus, the transcriptome analyses showed that MMRd-ins cancers are more likely than MMRd-del cancers to show elevated anti-cancer immunity, as evidenced by higher levels of immune cell infiltration and transcriptional upregulation of immune-related genes.

**Figure 5:**
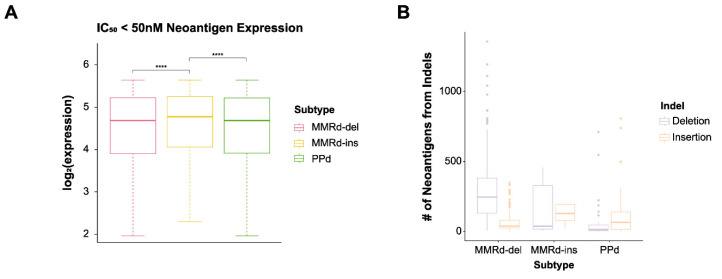

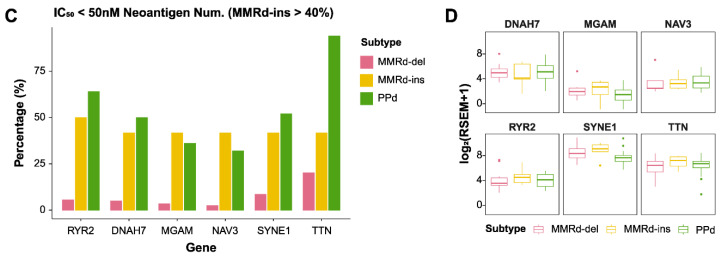
The number of neoantigens with strong HLA affinity. (**A**) High affinity (IC_50_ < 50 nM) neoantigens have high expression levels in MMRd-ins. *t*-test; **** *p* < 0.0001. (**B**) The number of neoantigens from indels. Neoantigens derived from deletions are most abundant in the MMRd-del subtype, whereas those from insertions are most abundant in the MMRd-ins subtype. Because the PPd subtype has fewer indels than the MMRd subtypes, its neoantigen abundance is the lowest. (**C**) Genes associated with IC_50_ < 50 nM neoantigens in more than 40% of MMRd-ins genomes. The PPd subtype has a ratio similar to that of the MMRd-ins subtype, whereas the MMRd-del subtype has a very low ratio. (**D**) The expression of six genes with high frequencies in the MMRd-ins subtype.

Elevated mutation rates in MMRd or PPd indel-enriched genomes lead to elevated rates of neoantigens, which can be associated with anticancer immunity. Therefore, we further investigated the abundance of neoantigens derived from indels or SNVs. Upon investigating neoantigens with high affinity (HLA-binding affinity IC_50_ < 50 nM), we observed significantly high expression levels in MMRd-ins genomes (Figure 5A; *t*-test; *p* = 4.4 × 10^20^ and *p* = 1.76 × 10^21^). We also observed that the abundance of neoantigens is largely proportional to the abundance of mutations (indels and SNVs), e.g., the abundance of neoantigens due to mutations in PPd genomes outnumbered those in MMRd genomes (MMRd-ins and MMRd-del) (Figure 5B and Figure S6A). MMRd-ins genomes had significantly more neoantigens derived from insertions than MMRd-del genomes due to their high frequency of insertions. The amount of expressed peptide-MHC (pMHC) from SNPs was significantly higher in PPd genomes, as was the number of immunogenic mutations (Figure S6B,C). Therefore, in PPd cancers, a high TMB might be associated with a more favorable prognosis due to enhanced immune activation [52,53]. When we investigated genes with high affinity, the MMRd-ins subtype exhibited notably high neoantigen frequency for the following genes: *RYR2*, *DNAH7*, *MGAM*, *NAV3*, *SYNE1*, and *TTN* (Figure 5C). Previously, *RYR2*, *DNAH7*, and *SYNE1* were shown to be associated with the immune response to various cancer types [54,55,56]. The expression of neoantigens for all of these genes except *DNAH7* was also higher in the MMRd-ins subtype (Figure 5D).

### 3.5. Genomic Signatures of MMRd-del and -ins Genomes Based on Whole Genome Sequencing

**Figure 6:**
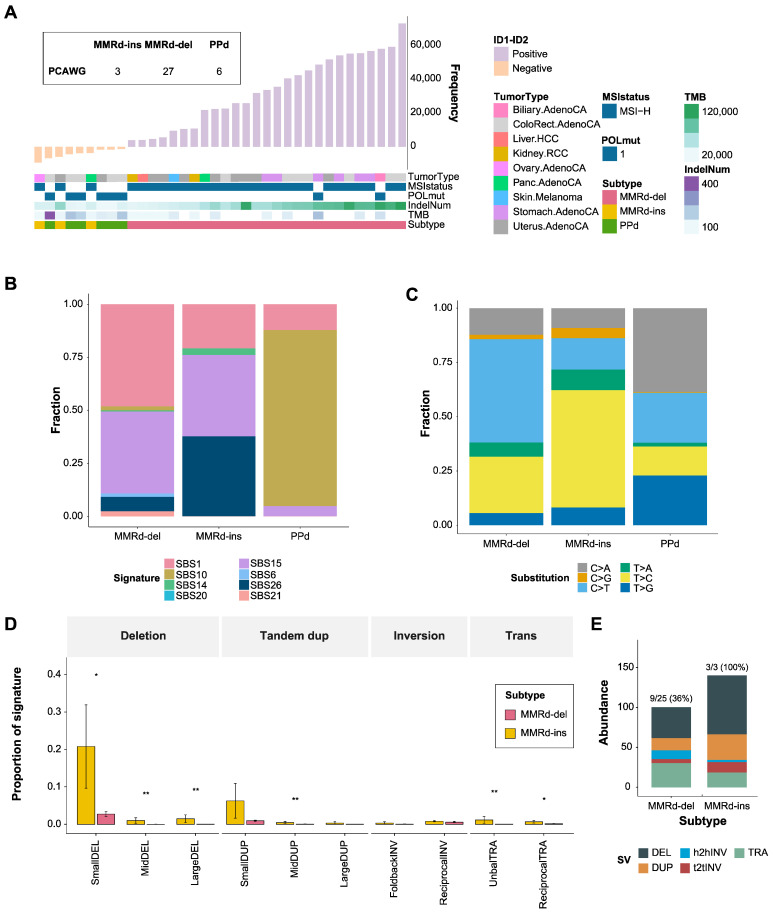
Validation of indel and SBS signatures using PCAWG data. (**A**) The top bar plot represents the indel frequency sorted by the difference in abundance between ID1 and ID2. Two samples with deletion-dominant *POLE* status were classified as MMRd-del. (**B**) The proportion of SBS signatures associated with MMRd and PPd. PPd has the highest proportion of SBS10 (brown), whereas MMRd-ins and MMRd-del are generally similar, with differences in the proportions of SBS1 (pink) and SBS26 (navy). (**C**) Ratio of single base substitutions in each subtype. PPd showed a difference in the C>A (gray) substitution rate, corresponding to SBS10, as compared with MMRd; and MMRd-ins exhibited differences in C>T (light blue) and T>C (dark blue) substitutions compared with MMRd-del. (**D**) Frequency of structural variant signatures in each subtype. Compared with the other subtypes, MMRd-ins showed a significantly higher frequency of small deletions and duplications. Wilcoxon test; * *p* < 0.05; ** *p* < 0.01. (**E**) The number of neoantigens from structural variants in MMRd-del and MMRd-ins.

We applied the strategy of indel-based tumor classification to whole genome sequencing datasets from PCAWG [57]. Among 2784 cancer genomes available with mutation profiles, we first selected 467 indel-enriched (>0.2 indels/Mb) genomes. Using ID1 and ID2 signature-related features (following the strategy in Figure 1A), these indel-enriched genomes were further discriminated into 27 MMRd-del, 3 MMRd-ins, and 6 PPd genomes (Figure 6A). We observed that MMRd-del, MMRd-ins and PPd annotation of the PCAWG genomes coincided with those of TCGA for the available cases: 1 MMRd-ins, 12 MMRd-del, and 2 PPd genomes. The detailed presentation of indel-related genomic features representing 1 bp deletion (ID2) and 1 bp insertion (ID1) are also shown in Figure S7A, and the cohort information is given in Table S4. Three MMRd-ins genomes were further verified to have an absence of *POLD1* and *POLE* mutations. Thus, the relative proportion of MMRd-ins genomes based on whole genome sequencing (10.0%, 3 MMRd-ins out of 30 MMRd ICGC genomes) is slightly higher, but largely consistent with that from whole exome sequencing (5.7%, 12 MMRd-ins out of 211 MMRd TCGA genomes).

The SBS mutation signatures from the whole genome sequencing–based mutation profiles are also largely consistent with the exome-based analysis results (Figure 2A). For example, the abundance of SBS26 and SBS1 significantly increased and decreased, respectively, in MMRd-ins genomes compared with MMRd-del genomes (Kruskal–Wallis test; *p* = 0.0013 and *p* = 5.6 × 10^5^, respectively), highlighting them as key distinguishing features between the two types of MMRd genomes (Figure 6B). A detailed presentation of SBS mutation signature abundance with statistics is available elsewhere (Figure S7B). Also consistent with the whole-exome results, T>C substitutions were highly increased in MMRd-ins, as shown in the subtype-wise mutation feature analysis (Figure 6C).

We further investigated the composition of SV types (deletion, inversion, and tandem duplication) and sizes (three bins: 0–100 kb as small, 100 kb–10 Mb as mid-sized, >10 Mb as large) for the three subtypes (Figure 6D). We noted that MMRd-ins genomes are relatively enriched with small/intermediate-sized deletions and duplications, compared with MMRd-del. The MMRd-ins subtype has a lower frequency of microhomology deletions than the MMRd-del subtype (Figure S1C), which suggests a potential for microhomology-mediated break-induced replication (MMBIR) events. The high frequency of small deletions and small tandem duplications in MMRd-ins could be associated with templated insertion mechanisms [38], suggesting a likelihood of an increase in insertion events. This implies the existence of a novel subtype with a mechanism distinct from the previously known MMR. We also observed neoantigens in most of the genomes exhibiting SVs (Figure 6E). Neoantigens were observed in all three MMRd-ins genomes but only nine of twenty-five MMRd-del genomes.

## 4. Discussion

Our study demonstrates that cancer genomes with MMRd can be distinguished into two distinctive subtypes based on the predominance of short insertions (MMRd-ins) or deletions (MMRd-del). We made this annotation using the ID1 and ID2 indel mutation signatures, and their corresponding features can be used to distinguish MMR-del and MMR-ins genomes, respectively. A previous report indicated that MMRd genomes often arise from genomes with a PPd deficiency [1], and we also observed that insertions are dominant in PPd genomes. However, our proposed new subtype, MMRd-ins, is characterized by the ID1 pattern with an absence of concurrent *POLE* and *POLD1* mutations. Although the proportion of MMRd-ins samples in the MSI-H cases was relatively low (5–10% in the exome- and genome-scaled mutation profiles), their consistent presence across a variety of tumor types suggests that they represent a distinct MSI subtype. This finding highlights the heterogeneity within MSI-H tumors and emphasizes the need for further investigation into the underlying mechanisms responsible for the emergence of the MMRd-ins subtype.

We also investigated the unique mutational characteristics of the MMRd-del, MMRd-ins, and PPd subtypes. Notably, in our analysis of SBS signatures, distinct SBS10 patterns characterized by frequent T[C>A]T and T[C>T]C substitutions were exclusively observed in PPd tumors, aligning with the recognized traits of tumors harboring POLE exonuclease mutations. Furthermore, discrepancies in the ratios of C>T (SBS1) and T>C (SBS26) substitutions were apparent between the MMRd-del and MMRd-ins subtypes, highlighting the genetic diversity within these MSI subtypes. Therefore, both ID and SBS features can be used to discriminate among the MSI subtypes. The SBS-based features of T>C preference were previously proposed to indicate the potential presence of MSI subtypes [58], but our indel-based analyses clearly document the presence of the MMRd-ins subtype.

We observed that MMRd-ins genomes can be highly immunogenic, with a higher level of immune cell infiltration and elevated immune-related functions compared with MMRd-del genomes. This could be attributed to the abundance of neoantigens derived from indels and SNPs in MMRd-ins, particularly those with high HLA-binding affinity in genes such as *RYR2*, *DNAH7*, and *SYNE1*. Those genes are involved in immune responses across various cancer types and could contribute to the enhanced recognition of MMRd-ins tumors by the immune system. Moreover, the upregulation of immune-related functional pathways, as revealed in our GSEA, further supports the notion of increased immune activity in MMRd-ins tumors. The elevated immune score reflects increased immune cell infiltration and suggests a greater likelihood of response to immunotherapy due to a suppressed tumor immune evasion phenotype.

Although we were not able to identify germline variants in the DDR pathway [59], we observed that the level of methylation and expression of the *MLH1* gene were diminished in MMRd-del and MMRd-ins genomes, albeit to a lesser extent in the latter. *MLH1* methylation and its concurrent transcriptional downregulation is a key somatic alteration leading to MSI [60,61]. The intermediate levels of *MLH1* methylation found in MMRd-ins genomes, compared with those in MMRd-del and PPd genomes, suggest that the MMRd-ins genomes might represent the early stages of MSI-H genomes, when *MLH1* methylation has not yet been fully acquired. Supporting that, we noted that the MMRd-ins subtype exhibits a significantly lower CCF than the other subtypes (Figure S4E). The observed intermediate level of *MLH1* methylation and low CCF levels thus suggest that MMRd-ins genomes might represent an early evolutionary phase of MSI in cancer genomes. The distinct mutational profiles between MMRd-ins and MMRd-del genomes further suggest that these two types of genomes might follow distinct evolutionary trajectories in acquiring their different mutational configurations.

Indels occur due to polymerase slippage errors during DNA replication. Most deletions arise from that mechanism [62,63,64]. However, the mechanism responsible for the preferred insertions over deletions in MMRd-ins genomes is not yet clearly understood. In the whole genome sequencing data, we found a unique pattern in the SV features of the MMRd-ins subtype, demonstrating the existence of a mechanism independent of MMR. Moreover, compared with MMRd-del genomes, MMRd-ins genomes are more likely to be associated with MMBIR accompanied by microhomology-related indel signature features (Figure S1C). Although significant frequency differences were not observed in pathogenic germline MMR genes (Figure S4A), the MMRd-ins genomes exhibited a higher frequency of mutations in the *PMS2* gene than the MMRd-del and PPd genomes. This is consistent with the previously presented increased insertion signature in *PMS2* [65], suggesting that it might induce insertion-dominant MSI.

Validation using the PCAWG dataset confirmed the consistency of subtype classification at both the exome and genome levels, affirming the robustness of our findings. The observed differences in SBS signatures between MMRd-del and MMRd-ins genomes were consistent across both datasets, providing further support for the feasibility of subtype classification based on ID signatures.

However, several limitations should be acknowledged. First, the relatively small sample size of the MMRd-ins subtype, especially when compared with the MMRd-del and PPd subtypes, limits the statistical power of our analyses and necessitates caution in drawing definitive conclusions. Future studies including a larger cohort may be required to validate our findings and generalize the conclusions. Second, our study lacks experimental validation. In vivo validation of the unique transcriptomics features and tumor behaviors such as the elevated immune cell infiltrations of MMRd-ins genomes might further support the clinical relevance of our results. In addition, we defined MSI-H annotations made by Bethesda criteria, primarily based on the instability in mononucleotide or dinucleotide microsatellite DNA sequences. We acknowledge the need to consider the potential presence of elevated microsatellite instability at selected tetranucleotide repeats (EMAST) tumors caused by isolated *MSH3* dysfunction [66]. Recent studies suggest that the EMAST genomes arise from stochastic frameshifting events in tetranucleotide repeats and also in di- or trinucleotide repeats, with concern that EMAST genomes may not represent a distinct cancer genomes subclass [67].

In conclusion, our comprehensive analysis of ID and SBS signatures, DNA methylation patterns, gene expression, immune-related pathways, and prognosis has identified distinct MSI subtypes. We have identified a novel subtype, MMRd-ins, characterized by unique mutational features distinct from those of both MMRd-del and PPd. These findings not only enhance our understanding of MSI but also have potential implications for personalized treatment strategies and prognostic assessments in MSI-associated cancers. Further research is warranted to elucidate the underlying mechanisms and clinical relevance of these MSI subtypes.

## Data Availability

The data presented in this study are available in Supplementary Materials.

## Supplementary Materials

The following supporting information can be downloaded at: https://www.mdpi.com/article/10.3390/genes15060770/s1, Figure S1. Landscape of indels. (A) Landscape of 83 indel signature features in all samples. (B) Tumor mutation and indel burden in ID1-dominant and ID2-dominant genomes. (C) Microhomology features of each subtype. Figure S2. SBS substitutions and signatures in each subtype. (A) Comparison of SBS signatures associated with MMRd and PPd. (B) Ratio of single-base substitutions in each subtype. Figure S3. Somatic mutation landscape of genes, as shown by Fisher’s exact test between MMRd-del and MMRd-ins genomes. (A) CRC (B) UCEC. Figure S4. Genomic alteration characteristics of each subtype. (A) Germline variant frequency of MMR-related genes and *POLE*/*POLD1*. (B) Genomic copy number alterations in CRC and (C) UCEC. (D) Cancer cell fraction of each subtype; Wilcoxon test; * *p* < 0.05; ** *p* < 0.01; *** *p* < 0.001; **** *p* < 0.0001. Figure S5. Immunity of each subtype. (A, B) TIDE score of tumor immune evasion in each subtype. (C) Immune cell fraction using CIBERSORTx and LM22 signature. Figure S6. Neoantigen abundance from SNVs. (A) The number of neoantigens with strong affinity (IC_50_ < 50 nM) from SNVs. (B) The number of expressed peptide-MHCs (pMHCs) from SNVs. (C) The number of immunogenic SNV neoantigens; Wilcoxon test; * *p* < 0.05; ** *p* < 0.01; *** *p* < 0.001; **** *p* < 0.0001. Figure S7. Landscape of indels and comparison of SBS signatures from PCAWG data. (A) Landscape of 83 indel signature features in all samples. (B) Comparison of SBS signatures associated with MMRd and PPd. Table S1. Abundance of all SBS signatures. Table S2. Cosine similarities for all SBS signatures across subtypes. Table S3. Gene set enrichment analysis (GSEA) results between MMRd-del and MMRd-ins genomes. Table S4. Indel-related genomic features representing 1bp deletions (ID2) and 1 bp insertions (ID1) in PCAWG data.
