# Supplementary figures and images for: A Subset of Microsatellite Unstable Cancer Genomes Prone to Short Insertions over Deletions Is Associated with Elevated Anticancer Immunity
Source: Genes (Basel). 2024 Jun 12;15(6):770. doi: 10.3390/genes15060770 (PMC11202581; doi:10.3390/genes15060770)

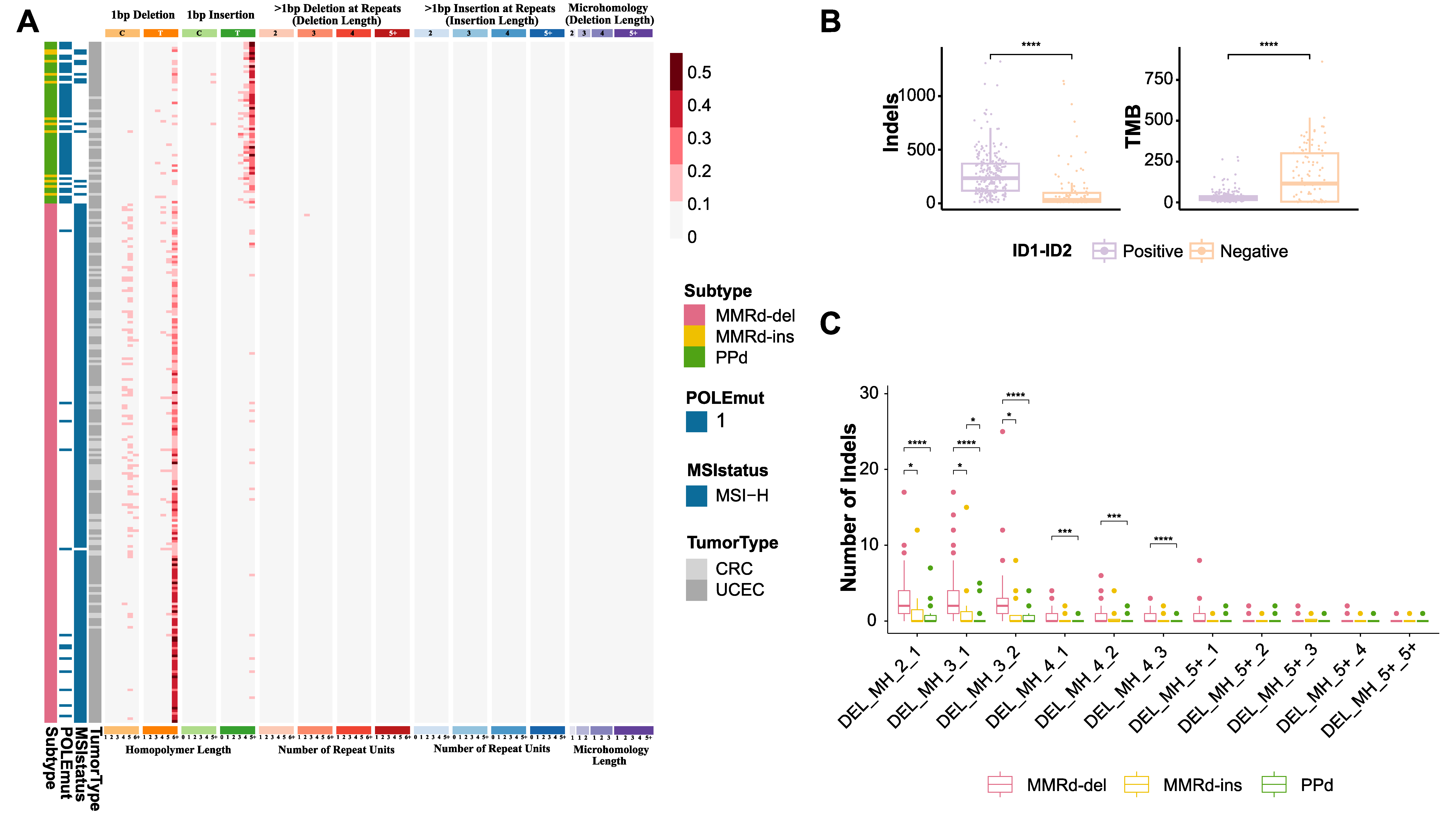

Supplement: Supplementary file 1 [file genes-15-00770-s001.zip › genes-3037846-supplementary/figure_s1.tif]

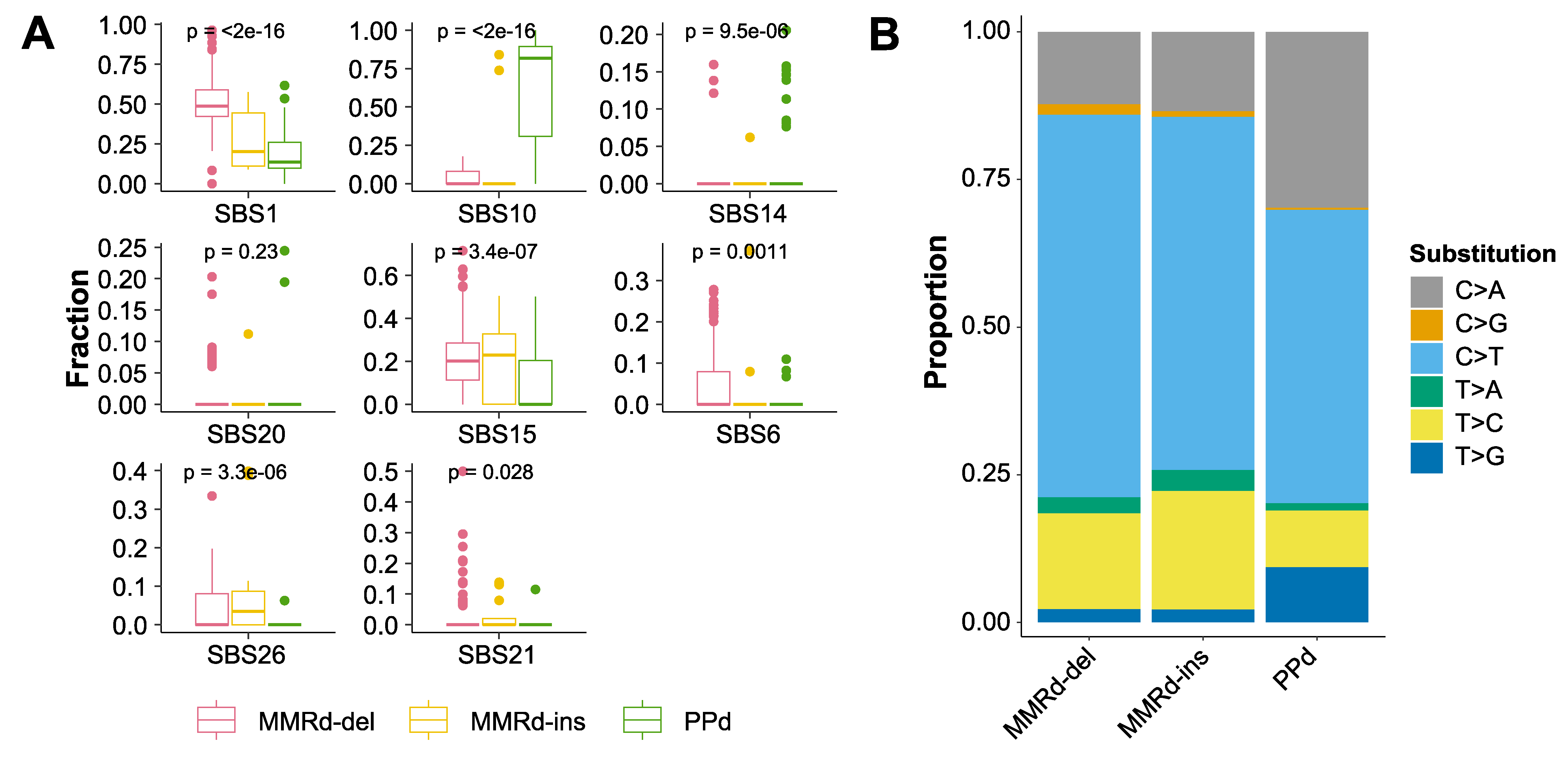

Supplement: Supplementary file 1 [file genes-15-00770-s001.zip › genes-3037846-supplementary/figure_s2.tif]

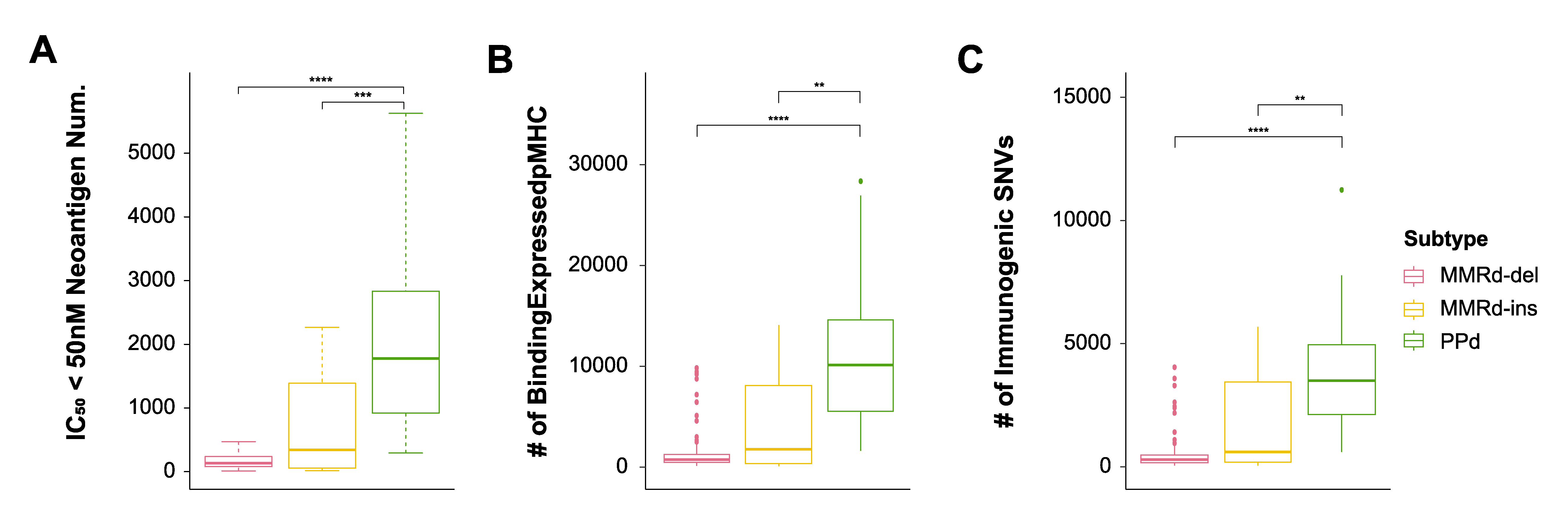

Supplement: Supplementary file 1 [file genes-15-00770-s001.zip › genes-3037846-supplementary/figure_s6.tif]

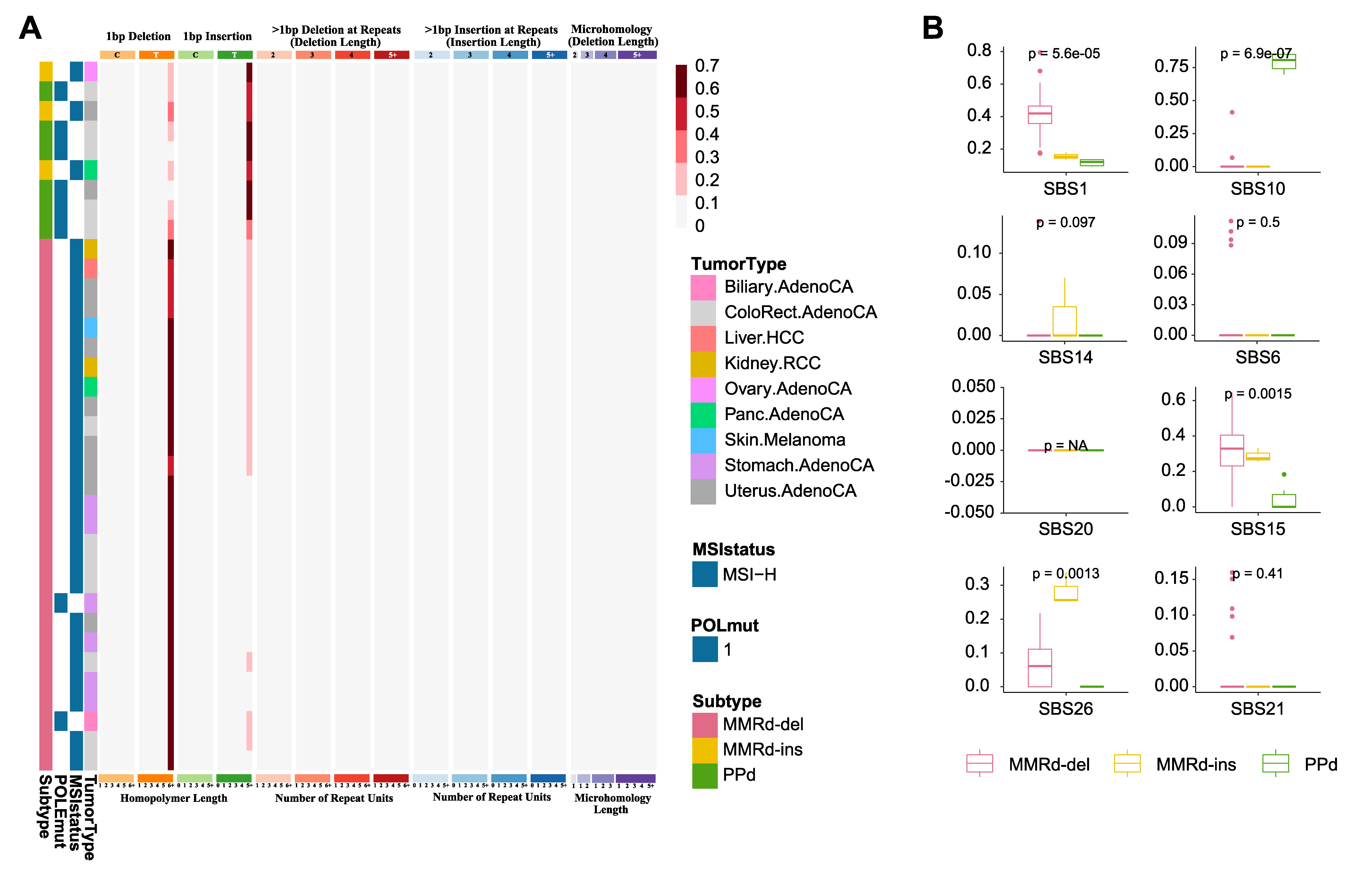

Supplement: Supplementary file 1 [file genes-15-00770-s001.zip › genes-3037846-supplementary/figure_s7.tif]
